# Oxygen uptake slow component: Enigma of the ‘excess’ oxygen used during heavy and severe exercise

**DOI:** 10.1113/EP092326

**Published:** 2024-10-28

**Authors:** David C. Poole, Glenn A. Gaesser

**Affiliations:** ^1^ Departments of Kinesiology, Anatomy and Physiology Kansas State University Manhattan Kansas USA; ^2^ College of Health Solutions Arizona State University Phoenix Arizona USA

**Keywords:** control of oxygen uptake, cycling biomechanics, heavy and severe exercise, muscle energetics, muscle fatigue, slow component

1

In the 1980s, foundational physiology and exercise physiology texts considered that oxygen uptake (${\dot{V}}_{O_{2}}$) increased as a linear function of work rate (WR) on the cycle ergometer. Whereas this was true up to the maximum ${\dot{V}}_{O_{2}}$ (${\dot{V}}_{O_{2}\max}$) for incremental exercise where WR was increased by 25 W or so per minute, for constant WR heavy‐ (i.e., >lactate threshold, LT) or severe‐ (>critical power, CP) intensity exercise there was an ‘excess’ ${\dot{V}}_{O_{2}}$ that manifested after the fast kinetics associated with the WR transition (Gaesser & Poole, 1996). This excess ${\dot{V}}_{O_{2}}$ became evident only after 2–3 min and for heavy exercise stabilized within 10 or so minutes or, for severe exercise, projected ${\dot{V}}_{O_{2}}$ to ${\dot{V}}_{O_{2}\max}$ auguring exhaustion shortly thereafter. In the extreme, this ${\dot{V}}_{O_{2}}$ slow component (${\dot{V}}_{O_{2}\mathrm{SC}}$) can amount to 1–1.5 L O_2_/min, eroding muscle efficiency.

For physiologists seeking to better understand muscle energetics and exercise limitations, non‐linear behaviour often provides powerful insights into systems control. However, at the time, explanations for the ${\dot{V}}_{O_{2}\mathrm{SC}}$ were less than satisfying. Typically, researchers in the field had extrapolated the estimated O_2_ cost of an array of physiological processes to calculate their putative role in the ${\dot{V}}_{O_{2}\mathrm{SC}}$ during heavy/severe intensity exercise (Gaesser & Poole, 1996). Alternatively, the temporal correlation of these processes with the ${\dot{V}}_{O_{2}\mathrm{SC}}$ was assessed to try and establish cause and effect. Principal processes considered included increasing ventilation, body temperature, blood lactate and catecholamines. Neither approach was especially satisfying; the former could explain far more than 100% of the ${\dot{V}}_{O_{2}\mathrm{SC}}$(!), and the latter could not discriminate causal from casual.

What was needed was a technique that had the power to parse among candidate variables. The initial strategy was to independently measure leg muscle(s) ${\dot{V}}_{O_{2}}$ simultaneously with pulmonary ${\dot{V}}_{O_{2}}$ during heavy/severe intensity exercise. This procedure unequivocally identified the exercising muscles as the source of the majority (>80%) of the ${\dot{V}}_{O_{2}\mathrm{SC}}$, thereby discounting a major role for ventilation, body temperature, auxiliary muscles and circulating factors such as lactate and catecholamines that might raise the metabolic rate outside the exercising muscles (Poole, 1994). Based upon subsequent a priori investigations, lactate and catecholamines as well as temperature increases acting within the exercising muscles were also discounted in this regard (Gaesser & Poole, 1996).

Viable remaining candidates relating to the altered physicochemical milieu associated with heavy/severe intensity exercise included fatiguing muscle fibres, recruitment of additional, more fast‐twitch, and less efficient muscle fibres, decreased mitochondrial P:O (i.e., ATP:O_2_) ratio and less efficient chemical–mechanical coupling. To date, most of these remain possibilities, though decreased P:O ratio – high O_2_ cost of phosphate production – seems unlikely (Rossiter et al., 2002) and, in dog and human muscles, the recruitment of additional muscle fibres is not requisite for a ${\dot{V}}_{O_{2}\mathrm{SC}}$ effect (e.g., Zoladz et al., 2008).

However, a tacit presumption of many previous investigators pursuing the mechanistic bases for the ${\dot{V}}_{O_{2}\mathrm{SC}}$ was that the external power produced reflected unchanged muscular input. One commendable novelty, among several, of a study by Macdougall and colleagues (2025), in this issue of *Experimental Physiology*, is that they considered and measured the biomechanics of cycling and determined how it changed relative to the generation of the ${\dot{V}}_{O_{2}\mathrm{SC}}$ in concert with (almost) real‐time determination of quadriceps muscle fatigue. Their data reveal that, although power remained constant over 20 min or so whilst the ${\dot{V}}_{O_{2}\mathrm{SC}}$ was developing, biomechanical indices of peak total downstroke force, minimum upstroke force and the upstroke index of effectiveness were not constant and showed a weak correlation with the ${\dot{V}}_{O_{2}\mathrm{SC}}$. Importantly, the observed association between muscle fatigue and the ${\dot{V}}_{O_{2}\mathrm{SC}}$ was highly significant, underlining the relevance of these findings despite considerable interindividual variability in the primary measurements. Indeed, even though all subjects exercised at a well‐defined power output of 10% above their individually estimated second lactate threshold, time to task failure ranged from ∼10 to 55 min. This is not trivial as factors affecting muscle fatigue as well as the ${\dot{V}}_{O_{2}\mathrm{SC}}$ undoubtedly differ when comparing fatiguing exercise among subjects with a >5‐fold range in time to task failure. The relatively high pedalling cadence of 80 rpm may have contributed to the large response heterogeneity as cadence has varying impacts on CP (i.e., relative to preferred cadence) (Carnevale & Gaesser, 1991). The metabolic stress of exercise may have differed across subjects as a result, making it more challenging to identify a specific cause for the associated ${\dot{V}}_{O_{2}}$ response across this substantial range in exercise duration.

The road from scientific discovery to general acceptance can be fraught with hurdles. For example, Dr Barbara McClintock's identification of ‘jumping genes’ in maize in the 1940s and 1950s was initially disbelieved, then begrudgingly acknowledged, but dismissed as unimportant. It was only decades later, in 1983, when its fundamental relevance was appreciated and rewarded with an unopposed Nobel Prize in Physiology or Medicine for the discovery of genetic transposition. For her brilliance, tenacity, and multiple apex achievements, she became, in 2005, the only woman scientist to be lauded with her picture on a U.S. postage stamp. In like fashion, the ${\dot{V}}_{O_{2}\mathrm{SC}}$ has been disputed, ignored, dismissed as unimportant or inconvenient, and until relatively recently, has been underappreciated for its great potential to resolve key facets of skeletal muscle energetics during exercise. The intriguing findings of MacDougall and colleagues (2025) take us away from the Ockham's razor approach of looking for a singular driver of the ${\dot{V}}_{O_{2}\mathrm{SC}}$ and force acknowledgment that not only may multiple factors contribute to its evolution, but those factors likely differ considerably across individuals.

Resolution of the mechanistic bases for the ${\dot{V}}_{O_{2}\mathrm{SC}}$ will not only enhance our fundamental understanding of muscle energetics but is also likely to have broad applications across medicine and society. For instance, if the ${\dot{V}}_{O_{2}\mathrm{SC}}$ can be controlled such that it does not progress the individual to ${\dot{V}}_{O_{2}\max}$, exercise tolerance can be increased. This is particularly pertinent for patients with heart failure or with chronic obstructive pulmonary disease (COPD, emphysema, chronic bronchitis) for whom the ${\dot{V}}_{O_{2}\mathrm{SC}}$ forces encroachment on their low and limiting ceiling for cardiovascular or ventilatory function, respectively, crippling exercise capacity (Gaesser & Poole, 1996). In contrast, with the upwards spiral of obesity in the developed world, it is conceivable that the ‘luxurious’ calorigenic effects of the ${\dot{V}}_{O_{2}\mathrm{SC}}$ could be marshalled to enhance the efficacy of exercise to restore a better caloric balance across affected populations. Specifically, targeting exercise that produced the greatest tolerable ${\dot{V}}_{O_{2}\mathrm{SC}}$ would expend more calories and thereby accelerate weight loss.

## AUTHOR CONTRIBUTIONS

All authors have read and approved the final version of this manuscript and agree to be accountable for all aspects of the work in ensuring that questions related to the accuracy or integrity of any part of the work are appropriately investigated and resolved. All persons designated as authors qualify for authorship, and all those who qualify for authorship are listed.

## CONFLICT OF INTEREST

None declared.

## References

[eph13689-bib-0001] Carnevale, T. J. , & Gaesser, G. A. (1991). Effects of pedaling speed on the power‐duration relationship for high‐intensity exercise. Medicine and Science in Sports and Exercise, 23, 242–246. 2017022

[eph13689-bib-0002] Gaesser, G. A. , & Poole, D. C. (1996). The slow component of oxygen uptake kinetics in humans. Exercise Sport Science Reviews, 24, 35–71. 8744246

[eph13689-bib-0003] MacDougall, K. B. , Aboodarda, S. J. , Westergard, P. H. , & MacIntosh, B. (2025). Muscle fatigue, pedalling technique and the V̇O* _2_ * slow component during cycling. Experimental Physiology, 110(1), 115–126. 10.1113/EP092116 PMC11689125 39412232

[eph13689-bib-0004] Poole, D. C. (1994). Role of exercising muscle in slow component of V̇O_2_ . Medicine Science Sports Exercise, 26, 1335–1340. 7837953

[eph13689-bib-0005] Rossiter, H. B. , Ward, S. A. , Howe, F. A. , Kowalchuk, J. M. , Griffiths, J. R. , & Whipp, B. J. (2002). Dynamics of intramuscular ^31^P‐MRS P(i) peak splitting and the slow components of PCr and O_2_ uptake during exercise. Journal of Applied Physiology (1985), 93, 2059–2069. 10.1152/japplphysiol.00446.2002 12391122

[eph13689-bib-0006] Zoladz, J. A. , Gladden, L. B. , Hogan, M. C. , Nieckarz, Z. , & Grassi, B. (2008). Progressive recruitment of muscle fibers is not necessary for the slow component of V̇O_2_ kinetics. Journal of Applied Physiology (1985), 105, 575–580. 10.1152/japplphysiol.01129.2007 18483168

